# Complete Genome Sequence of the Carotenoid-Producing *Deinococcus* sp. Strain AJ005

**DOI:** 10.1128/MRA.01245-19

**Published:** 2019-11-27

**Authors:** Jun Young Choi, Kunjoong Lee, Pyung Cheon Lee

**Affiliations:** a Department of Molecular Science and Technology, Ajou University, Suwon, South Korea; b Department of Applied Chemistry and Biological Engineering, Ajou University, Suwon, South Korea; Portland State University

## Abstract

The novel species *Deinococcus* sp. strain AJ005, isolated from King George Island, synthesizes a red carotenoid. Its complete genome is made up of a single circular chromosome (3,380,712 bp, 64.2% G+C content) and four circular plasmids.

## ANNOUNCEMENT

Some *Deinococcus* strains (1, 2) have attracted interest because they produce carotenoids of biotechnological significance and, therefore, have potential as microbial sources for carotenoids that can be used as cosmetic ingredients, antioxidants, and food or feed additives (3). A novel carotenoid-producing species, *Deinococcus* sp. strain AJ005, was isolated from seawater near the King Sejong Station in King George Island. Studying *Deinococcus* sp. strain AJ005 in detail, including whole-genome sequencing, in order to validate it as a potential carotenoid producer would be valuable. This genomic information provides a basis for developing recombinant *Deinococcus* strains and for elucidating carotenoid production mechanisms (4, 5) suitable for the large-scale fermentation of other biotechnologically important carotenoids.

The strain was obtained by single-colony isolation on tryptone-glucose-yeast (TGY) plates (tryptone, 5 g/liter; yeast extract, 5 g/liter; glucose, 1 g/liter; K_2_HPO_4_, 1 g/liter; agar, 20 g/liter; pH 7.0). The strain was aerobically cultured in TGY broth at 20°C for 5 days. Genomic DNA was extracted with a genomic DNA kit (Macrogen, South Korea) following the manufacturer’s protocols with RNase A treatment. Genomic DNA was sequenced on single-molecule real-time (SMRT) cells using PacBio RS II single-molecule real-time sequencing technology (Pacific Biosciences, CA) and with a HiSeq 2000 platform (Illumina, USA), both of which were operated by Macrogen, Inc. (Seoul, South Korea). The sequencing libraries were prepared using a DNA template prep kit v3.0 for PacBio RS II and a TruSeq Nano DNA kit for Illumina according to the manufacturer’s protocols. All software systems were run with their default settings unless otherwise noted. After subread filtering of the raw data, 125,025 reads with an average length of 10,128 bp (total, 1,266,349,440 bp), providing >300-fold genome coverage, were generated and *de novo* assembled using the Canu v1.3 assembler (6) with the setting “genomeSize=5m.” The overlapping regions at both ends of one contig were trimmed to make unique stretches on both ends using Circlator (7) with a parameter of “b2r_length_cutoff =60000, 100000, or 200000.” The resulting five assemblies were error corrected using Quiver in SMRT Analysis v2.3.0 (8) for four cycles. The error-corrected assemblies were further polished using Pilon v1.22 (9) (with the parameter “–fix bases”) with trimmed paired-end reads (total, 7,401,576 reads), which were obtained from 2 × 151-bp paired-end reads (total of 9,161,208 reads totaling 1,383,342,408 bp) using Sickle v1.33 (https://github.com/najoshi/sickle). The assembly statistics were calculated using stats.sh from BBmap v38.68 (https://sourceforge.net/projects/bbmap/). Genome annotation and gene prediction were performed using Rapid Annotations using Subsystems Technology (RAST) (10) with the ClassicRAST annotation scheme. Genes for rRNAs and tRNAs were predicted using RNAmmer v1.2 (11) and tRNAscan-SE v1.3.1 (12).

The genome of *Deinococcus* sp. strain AJ005 comprises a single 3,380,712-bp circular chromosome with a G+C content of 64.2%. There were four extrachromosomal circular plasmids (p380K, p115K, p96K, and p17K) present in *Deinococcus* sp. strain AJ005 (Table 1). Annotation revealed 3,295 coding DNA sequences and a total of 53 RNAs (three copies of the 16S, two copies of the 5S, and two copies of the 23S rRNA) on the 3.38-Mb chromosome. Notably, the 16S and 5/23S rRNA genes of *Deinococcus* sp. strain AJ005 are separated across the genome. Unlinked 16S and 5S/23S rRNA genes are common across *Deinococcus* species (13). Eight genes for carotenoid biosynthesis were predicted on the chromosome of *Deinococcus* sp. strain AJ005.

**TABLE 1:** Genome features of *Deinococcus* sp. strain AJ005

Genome element	Genome size (bp)	No. of contigs	G+C content (%)
Chromosome	3,380,712	1	64.2
p380K	380,021	1	64.2
p115K	115,048	1	60.0
p96K	96,101	1	64.8
p17K	17,648	1	52.4

### Data availability.

This whole-genome shotgun project has been deposited at DDBJ/EMBL/GenBank under the accession numbers CP044990 (chromosome), CP044988 (p17K), CP044987 (p96K), CP044991 (p115K), and CP044989 (p380K). The SRA/DRA/ERA accession numbers are SRX6953051 (Illumina) and SRX6955108 (PacBio). The BioSample and BioProject numbers are SAMN12911568 and PRJNA575821, respectively.
